# Predictors of unsuccessful tuberculosis treatment outcomes in Brazil: an analysis of 259,484 patient records

**DOI:** 10.1186/s12879-024-09417-7

**Published:** 2024-05-27

**Authors:** Do Kyung Ryuk, Daniele M. Pelissari, Kleydson Alves, Patricia Bartholomay Oliveira, Marcia C. Castro, Ted Cohen, Mauro Sanchez, Nicolas A. Menzies

**Affiliations:** 1 Department of Global Health and Population, Harvard T.H. Chan School of Public Health, 665 Huntington Ave, Boston, MA 02115 USA; 2 National Tuberculosis Programme, Ministry of Health, Brasilia, Brazil; 3 Pan-American Health Organization/World Health Organization, Brasilia, Brazil; 4 Department of Epidemiology of Microbial Diseases, Yale School of Public Health, New Haven, USA; 5 Department of Public Health, University of Brasilia, Brasilia, Brazil

## Abstract

**Introduction:**

Tuberculosis (TB) causes over 1 million deaths annually. Providing effective treatment is a key strategy for reducing TB deaths. In this study, we identified factors associated with unsuccessful treatment outcomes among individuals treated for TB in Brazil.

**Methods:**

We obtained data on individuals treated for TB between 2015 and 2018 from Brazil’s National Disease Notification System (SINAN). We excluded patients with a history of prior TB disease or with diagnosed TB drug resistance. We extracted information on patient-level factors potentially associated with unsuccessful treatment, including demographic and social factors, comorbid health conditions, health-related behaviors, health system level at which care was provided, use of directly observed therapy (DOT), and clinical examination results. We categorized treatment outcomes as successful (cure, completed) or unsuccessful (death, regimen failure, loss to follow-up). We fit multivariate logistic regression models to identify factors associated with unsuccessful treatment.

**Results:**

Among 259,484 individuals treated for drug susceptible TB, 19.7% experienced an unsuccessful treatment outcome (death during treatment 7.8%, regimen failure 0.1%, loss to follow-up 11.9%). The odds of unsuccessful treatment were higher with older age (adjusted odds ratio (aOR) 2.90 [95% confidence interval: 2.62–3.21] for 85-100-year-olds vs. 25-34-year-olds), male sex (aOR 1.28 [1.25–1.32], vs. female sex), Black race (aOR 1.23 [1.19–1.28], vs. White race), no education (aOR 2.03 [1.91–2.17], vs. complete high school education), HIV infection (aOR 2.72 [2.63–2.81], vs. no HIV infection), illicit drug use (aOR 1.95 [1.88–2.01], vs. no illicit drug use), alcohol consumption (aOR 1.46 [1.41–1.50], vs. no alcohol consumption), smoking (aOR 1.20 [1.16–1.23], vs. non-smoking), homelessness (aOR 3.12 [2.95–3.31], vs. no homelessness), and immigrant status (aOR 1.27 [1.11–1.45], vs. non-immigrants). Treatment was more likely to be unsuccessful for individuals treated in tertiary care (aOR 2.20 [2.14–2.27], vs. primary care), and for patients not receiving DOT (aOR 2.35 [2.29–2.41], vs. receiving DOT).

**Conclusion:**

The risk of unsuccessful TB treatment varied systematically according to individual and service-related factors. Concentrating clinical attention on individuals with a high risk of poor treatment outcomes could improve the overall effectiveness of TB treatment in Brazil.

**Supplementary Information:**

The online version contains supplementary material available at 10.1186/s12879-024-09417-7.

## Introduction

Tuberculosis (TB) is a major cause of infectious disease morbidity and mortality globally. In 2022, 1.3 million individuals are estimated to have died with TB, out of 10.6 million who developed incident TB [1]. Providing early diagnosis and effective treatment is a key strategy for reducing TB deaths. In the absence of drug resistance, TB is treated using a standardized 6-month course of rifampin, isoniazid, ethambutol, and pyrazinamide [2].

Following standardized reporting guidelines, the outcomes of TB treatment are categorized as one of five mutually-exclusive categories: cured, treatment completed, lost to follow-up, died, and treatment failed [3]. Treatment failure or loss to follow-up can result in a longer duration of disease, elevated mortality risks, and the possibility of acquired drug resistance [1]. To avoid these negative outcomes, it is important to implement effective patient-centric strategies to increase the fraction of patients achieving successful TB treatment outcomes [4].

Brazil is one of thirty high TB burden countries identified by the World Health Organization (WHO) [1]. In Brazil, TB diagnosis and treatment is provided through the universal healthcare system, Sistema Único de Saúde, under which TB incidence and mortality rates have decreased over time [5]. However, declines in TB incidence (which dropped from 54 to 43 per 100,000 between 2004 and 2014) stalled following an economic crisis in 2014 [6] and TB mortality was estimated as 3.3 per 100,000 in 2019 [7, 8]. During the COVID-19 pandemic, the national TB program reported an increase in loss to follow-up, from 11% in 2018 to 13% in 2020, and has also experienced decreasing rates of participation in directly observed therapy (DOT), from 38% in 2018 to 30% in 2020 [5]. In addition, the proportion of patients recorded as achieving cure has steadily decreased, from 73% in 2018 to 65% in 2020 [5]. By 2022 the TB mortality rate was estimated to have risen to 5.2 per 100,000 [1].

Understanding how different factors are associated with TB treatment outcomes can suggest approaches to improving care, and help identify patients with the greatest risks of experiencing unsuccessful outcomes. In this study, we assessed potential risk factors for unsuccessful TB treatment outcomes under routine clinical conditions in Brazil’s national TB treatment program. Using national disease registry data, we analyzed treatment outcomes for individuals initiating TB treatment between 2015 and 2018. We estimated how treatment outcomes varied by demographic and socio-economic factors, the presence of co-morbidities, health-related behaviors, and features of service provision, as well as how outcomes varied across Brazilian states.

## Method

### Data sources

We conducted a cross-sectional study, in which we obtained data on all individuals with notified TB disease between 2015 and 2018 (*n* = 356,119) from Brazil’s National Disease Notification Information System (SINAN: Sistema de Informação de Agravos de Notificação). These data record final treatment outcomes for individuals diagnosed with TB, including pulmonary and extrapulmonary disease, in all 26 Brazilian states and the Federal District (Brasília).

We excluded patients with a history of previous TB treatment (*n* = 68,519, 19.2%), patients diagnosed with resistance to rifampicin (*n* = 2,584, 0.7%), patients who had a change in regimen due to adverse event or identified drug-resistance (*n* = 2,019, 0.6%), patients transferred to a different provider during therapy (*n* = 20,306, 5.7%), patients diagnosed with TB post-mortem (*n* = 2,695, 0.8%), patients with a missing value for treatment outcome (*n* = 10,786, 3.0%), and patients with illogical values for exposure variables, such as miscategorized age (*n* = 56, < 0.1%) [9]. For each individual included in the study cohort we extracted information on patient-level factors potentially associated with TB treatment outcomes. These include socio-demographics (sex, age, education, self-declared race), vulnerability status (incarcerated, homelessness, immigrants), other health conditions (HIV, diabetes), health-related behaviors (illicit drug use, alcohol consumption, current smoking), type of TB disease (pulmonary, extrapulmonary, or both), aspects of clinical care (participation in DOT, pre-treatment diagnostic test results (bacteriological diagnosis, chest x-ray)), and the health system level at which treatment was provided (obtained through linkage between SINAN and the National Registry of Health Establishment (CNES)). Table 1 provides definitions for each outcome and exposure variable. We also recorded the state in which each individual received treatment.

**Table 1:** Definitions of outcome and exposure variables

Variable	Variable definition
**Outcome variables**	
Unsuccessful treatment outcome *(main analysis)*	Yes (includes loss to follow-up, died, treatment failure), No (includes treatment completion, cure).
Categorical treatment outcome *(secondary analysis)*	Died, loss to follow-up, success (includes completion, cure).*
**Exposure variables**	
Age group	Difference between patient’s notification date and their recorded date of birth, in years. Categorized as 0–4, 5–14, 15–24, 25–34, 35–44, 45–54, 55–64, 65–74, 75–84, or 85–100 years.
Sex	Categorized as male or female.
Education level	Highest level of education attained, categorized as no education, incomplete/complete 1-4th grade, complete 5-8th grade, complete high school education, any higher education, or other.
Race	Categorized as ‘White’, ‘Black’, ‘Yellow’, ‘Mixed’, ‘Indigenous’, or ‘Other’, which represent the race categories recorded in SINAN.
HIV	Whether patient is living with HIV, categorized as yes, no, or other.
Diabetes	Whether patient has diagnosed diabetes. Categorized as yes, no, or other.
Illicit drug use	Whether patient reported using illicit drugs, categorized as yes, no, or other.
Alcohol use	Whether patient reported using alcohol, categorized as yes, no, or other.
Smoking	Whether patient reported smoking, categorized as yes, no, or other.
Incarcerated	Whether patient was incarcerated at time of diagnosis, categorized as yes, no, or other.
Homeless	Whether patient was homeless at time of diagnosis, categorized as yes, no, or other.
Immigrant	Whether patient reported being an immigrant, categorized as yes, no, or other.
Level of health service	Level of care of the facility at which TB treatment was provided, categorized as primary, secondary, tertiary, or other.
Received DOT	Whether TB treatment was provided via DOT, categorized as yes, no, or other.
Bacteriological test result	Whether patient had bacteriologically-confirmed TB (i.e., via sputum smear microscopy, culture, or Xpert MTB-RIF) at diagnosis, categorized as positive, negative, or not determined.
Chest x-ray result Type of TB	Interpretation of chest x-ray taken as part of diagnosis, categorized as presumed with TB, normal, or not performed. Determined as part of diagnosis, categorized as pulmonary, extrapulmonary or both.

DOT = directly observed therapy. HIV = human immunodeficiency virus. “Other” category for HIV variable refers to patients who have their test in progress, or patients who did not test. “Other” category for variables including diabetes, illicit use of drugs, alcohol, smoking, DOT, patients in vulnerable circumstances (incarcerated, homeless, immigrant) includes patients who did not respond to the question or who otherwise had missing values. “Other” category for the level of health service variable includes laboratory centers or private clinics. * Analyses of this secondary outcome excluded individuals with a treatment outcome not falling into one of the categories shown

### Outcome definition

In SINAN, individuals treated for TB can have a treatment outcome recorded as ‘treatment success’, representing the sum of ‘cured’ (defined as initially smear-positive individuals with at least two successive negative sputum smears before completing treatment) and ‘treatment completed’ (defined as initially smear-negative individuals completing treatment with no positive smears and no clinical or radiological evidence of failure) treatment outcome categories [3]. Individuals recorded with ‘death on treatment’ (defined as death from TB or other cause during TB treatment), ‘regimen failure’ (defined as having positive sputum smear or culture in the 4th month or two consecutive months after the 4th month of treatment initiation) or ‘loss to follow-up’(defined as the patient not attending the treatment facilities for 30 days or more once treatment has started) were coded as having an unsuccessful treatment outcome [10].

For the main analysis we analyzed a binary outcome indicating whether the individual experienced an unsuccessful treatment outcome. As a secondary analysis we analyzed a categorical outcome with three levels (treatment success, loss to follow-up, and death) to allow for different predictors of loss to follow-up and death. For this secondary analysis, we did not consider the outcome of treatment failure, given the small number of individuals in this group.

### Statistical analysis

We fitted univariate and multivariate logistic regression models to identify factors associated with unsuccessful treatment outcomes, considering each exposure variable as well as state of residence. For most variables we selected the category with the highest number of observations as the reference group. For race and education level, we selected ‘White’ and ‘completed high-school education’ (respectively) as the reference categories, representing population groups historically associated with better TB outcomes, such that the results describe the excess risks faces by other populations. Results are reported as odds ratios. For the secondary analysis of categorical treatment outcome (success, loss to follow-up, death), we fitted multinomial logistic regression models to estimate the factors associated with specific treatment outcomes, with results reported as relative risk ratios. As a sensitivity analysis, we refit separate regression models for the binary treatment outcome to data for each calendar year.

We conducted additional analyses to estimate the importance of each exposure variable in explaining treatment outcomes within the study cohort. To do so, we refit the main analysis regression model (for the binary treatment outcome) excluding each covariate one at a time, and estimated Akaike Information Criterion (AIC) for each of these models. We calculated the difference between these values and the AIC estimated for the full model including all the covariates, reporting these difference measures as an indicator of variable importance. We calculated confidence intervals for these results using a bootstrap approach with 1000 replicates. All analyses were conducted in R [10].

## Results

Table 2 describes the distribution of individuals across levels of each exposure variable. Among 259,484 individuals included in the study cohort, 19.7% (*n* = 51,160) experienced an unsuccessful treatment outcome (death on treatment 7.8%, regimen failure 0.1%, loss to follow-up 11.9%).

**Table 2:** Baseline information and treatment outcomes for the study population

Variable (category)		Total sample	Unsuccessful treatment	Variable (category)		Total sample	Unsuccessful treatment
**Age group**	0–4	3,018	406	**Alcohol**	Yes	41,723	12,805
	5–14	4,841	433		No	202,673	34,232
	15–24	64,688	8,418		Other	15,088	4,123
	25–34	49,267	11,316	**Drug**	Yes	30,380	10,250
	35–44	47,682	10,119		No	209,783	35,689
	45–54	39,913	7,924		Other	19,321	5,221
	55–64	30,697	5,921	**Incarcerated**	Yes	24,882	3,276
	65–74	15,884	3,616		No	218,693	44,057
	75–84	7,363	2,187		Other	15,909	3,827
	85+	1,994	820	**Homeless**	Yes	6,172	3,363
**Sex**	Male	177,330	38,002		No	236,215	43,824
	Female	82,160	13,158		Other	17,097	3,973
**Race**	White	82,426	14,377	**Immigrants**	Yes	1,543	371
	Black	31,404	7,245		No	236,979	46,021
	Yellow	1,825	323		Other	20,962	4,768
	Mixed	122,125	24,604	**Health unit**	Primary care	140,807	21,139
	Indigenous	2,909	426		Seconary care	74,173	16,232
	Other	18,795	4,185		Tertiary care	35,854	12,260
**Education**	No education	11,179	2,634		Other	8,650	1,529
	Incomplete 1-4th grade	29,434	6,215	**DOT**	Yes	99,353	10,445
	Complete 1-4th grade	60,779	12,560		No	97,422	21,560
	Complete 5-8th grade	47,932	7,965		Other	62,709	19,155
	Complete high school	24,137	3,165	**Bacteriological**	Positive	169,512	31,569
	Any higher education	16,596	1,622	**test**	Negative	44,195	9,178
	Other	69,427	16,999		Not determined	45,777	10,413
**Diabetes**	Yes	19,937	3,621	**Chest X-ray**	Suggestive	183,784	37,260
	No	223,818	43,193		Normal	16,977	3,177
	Other	15,729	4,346		Not performed	58,723	10,723
**HIV**	Yes	23,328	9,501	**Type of TB**	Pulmonary	217,486	174,675
	No	191,119	28,832		Extrapulmonary	7,500	5,282
	Other	45,037	12,827		Both	34,498	28,367
**Smoking**	Yes	53,390	13,790				
	No	188,028	32,424				
	Other	18,066	4,946				

DOT = directly observed therapy. HIV = human immunodeficiency virus

### Odds ratios for unsuccessful treatment

Unadjusted and adjusted odds ratios for unsuccessful treatment for each exposure variable are reported in Table 3, based on the results of univariate and multivariate regression models, respectively. Significant differences in the odds of unsuccessful treatment were estimated for several exposure variables. We estimated elevated risks of unsuccessful treatment (adjusted odds ratios (aORs) > 1.0) for variables describing age > 65 years (versus age 25–34), Black race (versus White race), educational level less than complete high school education (versus complete high school education), HIV-positive or HIV unknown status (versus HIV-negative), smoking (versus non-smoking), alcohol consumption (versus no alcohol consumption), illicit drug use (versus no illicit drug use), homelessness (versus no homelessness), immigrant status (versus non-immigrants), treatment provision in secondary or tertiary care (versus primary care), not enrolled in DOT therapy (versus DOT), bacteriological test negative or not determined (versus individuals with a positive bacteriological test result), and chest x-ray not performed (versus x-ray suggestive of TB). Age < 15 years (versus age 25–34), female sex (versus male sex), education above high school level (versus complete high school education), diabetes (versus no diabetes), and incarceration (versus non incarceration) were associated with lower risks of unsuccessful treatment.

**Table 3:** Raw and adjusted odds ratio for unsuccessful treatment for each exposure variable, 2015–2018

Variables (reference category)	Univariate odds ratio (95% CI)	Adjusted odds ratio (95% CI)
**Age (25–34)**		
0–4	0.65 (0.59, 0.73)	0.50 (0.45, 0.56)
5–14	0.41 (0.37, 0.46)	0.39 (0.35, 0.43)
15–24	0.87 (0.84, 0.90)	1.04 (1.01, 1.08)
35–44	1.13 (1.10, 1.17)	0.93 (0.90, 0.97)
45–54	1.04 (1.01, 1.07)	0.91 (0.88, 0.94)
55–64	1.00 (0.97, 1.04)	0.98 (0.94, 1.02)
65–74	1.24 (1.19, 1.29)	1.25 (1.19, 1.31)
75–84	1.77 (1.68, 1.87)	1.90 (1.79, 2.02)
85+	2.93 (2.68, 3.21)	2.90 (2.62, 3.21)
**Sex (Male)**		
Female	0.70 (0.68, 0.71)	0.78 (0.76, 0.80)
**Race (White)**		
Black	1.42 (1.37, 1.47)	1.23 (1.19, 1.28)
Yellow	1.01 (0.90, 1.15)	0.99 (0.87, 1.12)
Mixed	1.19 (1.17, 1.22)	1.14 (1.11, 1.12)
Indigenous	0.81 (0.73, 0.90)	1.05 (0.94, 1.17)
Other	1.36 (1.30, 1.41)	1.02 (0.98, 1.07)
**Education (complete high school)**		
No education	2.04 (1.93, 2.16)	2.03 (1.91, 2.17)
Incomplete 1-4th grade	1.77 (1.69, 1.86)	1.80 (1.71, 1.90)
Complete 1-4th grade	1.73 (1.65, 1.80)	1.83 (1.74, 1.91)
Complete 5-8th grade	1.32 (1.26, 1.38)	1.46 (1.39, 1.53)
Any higher education	0.72 (0.67, 0.76)	0.84 (0.79, 0.90)
Other	2.15 (2.06, 2.24)	1.94 (1.85, 2.03)
**Diabetes (no)**		
Yes	0.93 (0.89, 0.96)	0.91 (0.87, 0.94)
Other	1.60 (1.54, 1.66)	0.90 (0.85, 0.97)
**HIV (no)**		
Yes	3.87 (3.76, 3.98)	2.72 (2.63, 2.81)
Other	2.24 (2.19, 2.30)	1.83 (1.78, 1.88)
**Smoking (no)**		
Yes	1.67 (1.63, 1.70)	1.20 (1.16 1.23)
Other	1.81 (1.75, 1.87)	1.10 (1.02, 1.18)
**Alcohol (no)**		
Yes	2.18 (2.13, 2.23)	1.46 (1.41, 1.50)
Other	1.85 (1.78, 1.92)	1.09 (1.01, 1.17)
**Illicit drug use (no)**		
Yes	2.48 (2.42, 2.55)	1.95 (1.88, 2.01)
Other	1.81 (1.75, 1.87)	1.21 (1.13, 1.30)
**Incarcerated (no)**		
Yes	0.60 (0.58, 0.62)	0.52 (0.49, 0.54)
Other	1.26 (1.21, 1.30)	1.18 (1.05, 1.32)
**Homeless (no)**		
Yes	5.26 (4.99, 5.53)	3.12 (2.95, 3.31)
Other	1.33 (1.28, 1.38)	0.87 (0.77, 0.99)
**Immigrants (no)**		
Yes	1.31 (1.17, 1.48)	1.27 (1.11, 1.45)
Other	1.22 (1.18, 1.26)	0.92 (0.85, 1.00)
**Health unit (primary care)**		
Secondary care	1.59 (1.55, 1.62)	1.20 (1.17, 1.24)
Tertiary care	2.94 (2.87, 3.02)	2.20 (2.14, 2.27)
Other	1.22 (1.15, 1.29)	1.02 (0.95, 1.08)
**DOT (yes)**		
No	2.42 (2.36, 2.48)	2.35 (2.29, 2.41)
Other	3.74 (3.65, 3.84)	3.13 (3.04, 3.22)
**Bacteriological test (positive)**		
Negative	1.15 (1.12, 1.18)	1.14 (1.11, 1.18)
Not determined	1.29 (1.26, 1.32)	1.33 (1.29, 1.38)
**Chest X-ray (suggestive of TB)**		
Normal	0.91 (0.87, 0.94)	0.95 (0.91, 1.00)
Not performed	0.88 (0.86, 0.90)	1.03 (1.00, 1.06)
**Type of TB (pulmonary)**		
Extrapulmonary	1.71 (1.63, 1.80)	1.03 (0.98, 1.09)
Both	0.88 (0.86, 0.91)	0.72 (0.69, 0.75)

DOT = directly observed therapy. HIV = human immunodeficiency virus. CI = confidence interval. Raw odds ratios estimated from regression models including each exposure variable individually. Adjusted odds ratios estimated from a regression model including all exposure variables

For most exposure variables univariate ORs were similar to the results of the multivariate analysis. However, univariate ORs for HIV, smoking, alcohol consumption, illicit drug use, and homelessness were elevated compared to adjusted ORs, consistent with clustering of these risk factors within a subset of patients experiencing worse treatment outcomes. In sensitivity analyses we refit separate regression models to the data for each calendar year (Table S2). These results were generally similar to those estimated in the main analysis.

### State-level differences in treatment outcome

**Fig. 1:**
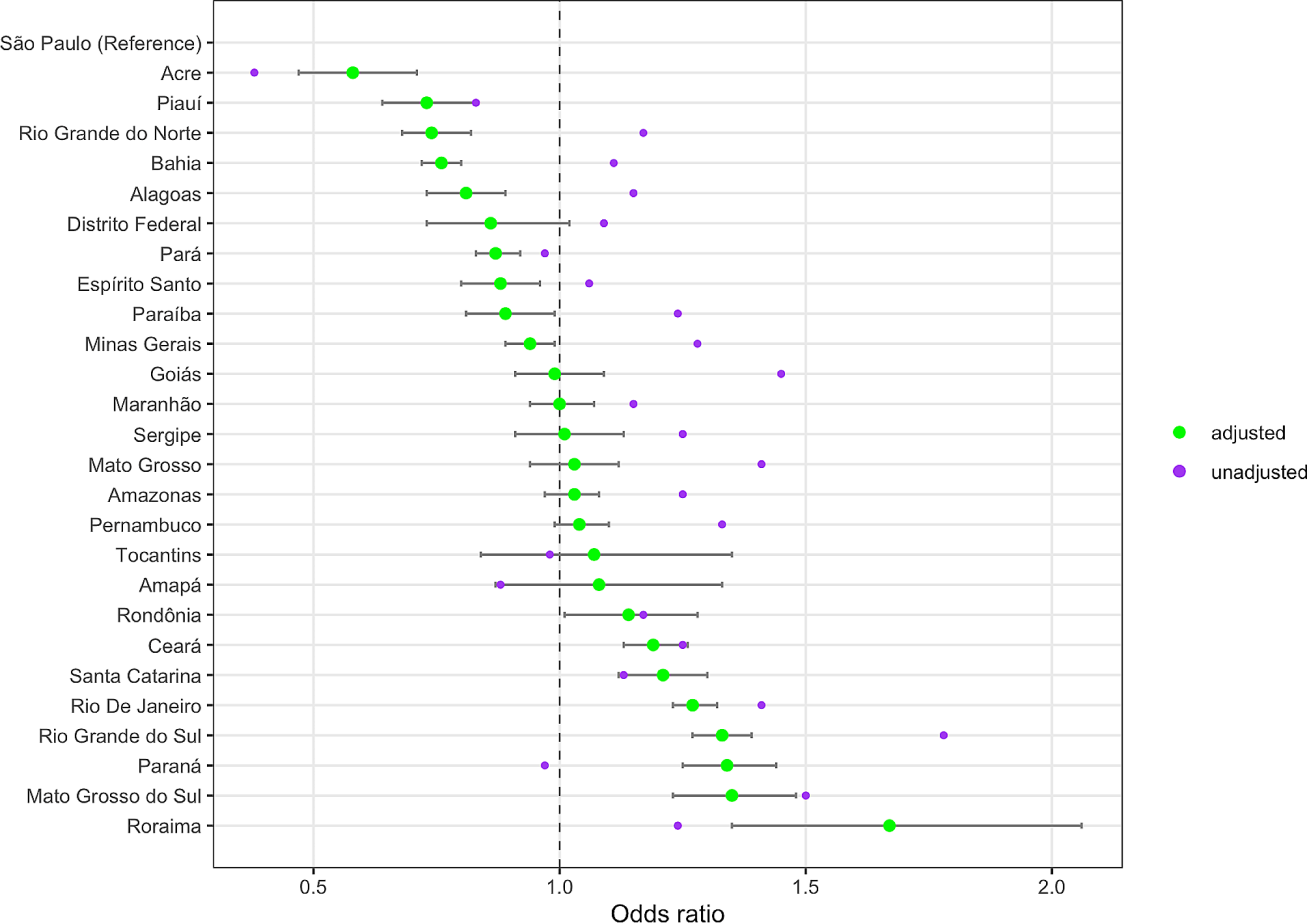
Unadjusted and adjusted odds ratios of unsuccessful treatment for each state, 2015–2018. Unadjusted odds ratios estimated from regression models including each exposure variable individually. Adjusted odds ratios estimated from a regression model including all exposure variables. Horizontal bars represent 95% confidence intervals for adjusted odds ratios

At the state level, the univariate model described the highest odds of unsuccessful treatment in the state of Rio Grande do Sul (OR = 1.78, 95% CI: 1.71–1.85), and the lowest odds of unsuccessful treatment in the state of Acre (OR = 0.38, 95% CI: 0.31–0.47), both compared to the state of São Paulo (Fig. 1, Table S1). Adjusted odds ratios (controlling for all other exposure variables) described the highest odds of unsuccessful treatment in the state of Roraima (aOR = 1.67, 95% CI: 1.35–2.06), and the lowest odds in the state of Acre (aOR = 0.58, 95% CI: 0.47–0.71).

### Importance of individual exposure variables and sensitivity analysis

Table 4 presents results describing the relative importance of each exposure variable. Based on these results, treatment via DOT, HIV status, healthcare level of the treatment provider, education level, and age group were the most important variables in terms of explaining the variation in treatment outcomes within the study cohort.

**Table 4:** Importance of each exposure variable for explaining cohort treatment outcomes

Exposure variable	Variable importance* (95% CI)
Chest x-ray result	5 (-2, 23)
Diabetes	11 (0, 32)
Immigrant status	16 (3, 40)
Smoking status	136 (91, 192)
Race	214 (163, 280)
Bacteriological test result	329 (266, 410)
Type of TB	343 (276, 426)
Sex	363 (297, 445)
Alcohol use	517 (419, 611)
Incarceration	912 (799, 1,034)
Illicit drug use	1,473 (1,322, 1,634)
Homeless	1,523 (1,362, 1,680)
Age group	1,661 (1,506, 1,833)
Education level	1,731 (1,571, 1,902)
Health care level of treatment provider	2,506 (2,317, 2,715)
HIV status	4,906 (4,619, 5,169)
DOT received	7,294 (6,965, 7,622)

DOT = directly observed therapy. HIV = human immunodeficiency virus. Variable importance calculated from the difference in AIC for models excluding each exposure variable as compared to the full regression model for the binary treatment outcome (AIC = 222,461). Greater values indicate greater importance for a given exposure variable

### Risk ratios for categorical outcome

Table 5 presents results for the categorical treatment outcome (success, death, loss to follow-up) estimated via multinomial logistic regression. For several variables the factors associated with loss to follow-up differ from those associated with death on treatment. Age > 35 years, diabetes, ‘Other’ care provider, and extrapulmonary TB were each associated with higher risks of death and lower risks of loss to follow-up. Conversely, age 15-24-years, ‘Other’ race, and x-ray not performed were associated with lower risks of death and higher risks of loss to follow-up. Black or Mixed race, lower education levels (less than complete high school), HIV, presence of behavioral risk factors (smoking, alcohol use, illicit drug use), homelessness, tertiary care, treatment not provided via DOT, and not determined bacteriological test result were all associated with higher risks of both loss to follow-up and death. Lower risks for both negative outcomes were estimated for age < 15 years, female sex, any higher education, unknown diabetes status, incarceration, and TB with pulmonary involvement.

**Table 5:** Adjusted relative risk ratios for categorical treatment outcome, 2015–2018

Variables (reference)	Adjusted RR (95% CI)	
	Loss to follow-up	Death
**Age (25–34)**		
0–4	0.50 (0.44, 0.58)	0.71 (0.59, 0.84)
5–14	0.47 (0.42, 0.53)	0.36 (0.29, 0.45)
15–24	1.11 (1.07, 1.15)	0.74 (0.69, 0.80)
35–44	0.86 (0.83, 0.89)	1.34 (1.27, 1.42)
45–54	0.62 (0.60, 0.65)	1.95 (1.84, 2.06)
55–64	0.45 (0.43, 0.48)	2.94 (2.77, 3.12)
65–74	0.39 (0.36, 0.42)	4.66 (4.37, 4.98)
75–84	0.55 (0.41, 0.51)	7.42 (6.87, 8.01)
85+	0.80 (0.45, 0.67)	11.83 (10.54, 13.28)
**Sex (Male)**		
Female	0.80 (0.77, 0.82)	0.80 (0.77, 0.82)
**Race (White)**		
Black	1.40 (1.34, 1.46)	1.08 (1.03, 1.14)
Yellow	1.20 (1.03, 1.40)	0.86 0.70, 1.06)
Mixed	1.18 (1.15, 1.22)	1.04 (1.01, 1.08)
Indigenous	0.99 (0.86, 1.14)	0.91 (0.77, 1.07)
Other	1.10 (1.04, 1.16)	0.87 (0.81, 0.93)
**Education (Complete high school)**		
No education	1.95 (1.80, 2.12)	2.03 (1.86, 2.22)
Incomplete 1-4th grade	1.78 (1.67, 1.89)	1.75 (1.62, 1.89)
Complete 1-4th grade	1.82 (1.72, 1.92)	1.54 (1.43, 1.65)
Complete 5-8th grade	1.47 (1.39, 1.55)	1.25 (1.16, 1.35)
Any higher education	0.78 (0.72, 0.85)	0.78 (0.70, 0.86)
Other	1.74 (1.65, 1.84)	2.07 (1.93, 2.22)
**Diabetes (no)**		
Yes	0.81 (0.76, 0.86)	1.10 (1.05, 1.16)
Other	0.98 (0.91, 1.06)	0.90 (0.82, 0.99)
**HIV (no)**		
Yes	1.92 (1.84, 2.00)	5.09 (4.87, 5.32)
Other	1.91 (1.85, 1.97)	1.79 (1.72, 1.86)
**Smoking (no)**		
Yes	1.22 (1.18, 1.26)	1.11 (1.07, 1.16)
Other	1.05 (0.96, 1.15)	1.26 (1.14, 1.39)
**Alcohol (no)**		
Yes	1.36 (1.31, 1.41)	1.59 (1.52, 1.66)
Other	1.01 (0.92, 1.10)	1.09 (0.98, 1.20)
**Illicit drug use (no)**		
Yes	2.19 (2.11, 2.27)	1.29 (1.21, 1.37)
Other	1.18 (1.09, 1.29)	1.19 (1.08, 1.31)
**Incarcerated (no)**		
Yes	0.54 (0.52, 0.57)	0.44 (0.40, 0.48)
Other	0.95 (0.83, 1.09)	1.04 (0.86, 1.24)
**Homeless (no)**		
Yes	3.82 (3.59, 4.06)	2.11 (1.93, 2.31)
Other	0.90 (0.78, 1.04)	0.88 (0.73, 1.07)
**Immigrants (no)**		
Yes	1.43 (1.24, 1.66)	1.19 (0.96, 1.48)
Other	1.11 (1.02, 1.22)	1.00 (0.89, 1.13)
**Health unit (primary care)**		
Secondary care	1.01 (0.98, 1.04)	1.74 (1.67, 1.82)
Tertiary care	1.24 (1.19, 1.29)	4.46 (4.28, 4.66)
Other	0.81 (0.76, 0.88)	1.60 (1.46, 1.75)
**DOT (yes)**		
No	3.06 (2.96, 3.16)	1.56 (1.49, 1.63)
Other	3.43 (3.31, 3.56)	2.88 (2.76, 3.00)
**Bacteriological test (positive)**		
Negative	1.00 (0.96, 1.04)	1.43 (1.37, 1.50)
Not determined	1.16 (1.12, 1.21)	1.68 (1.61, 1.75)
**Chest X-ray (suggestive with TB)**		
Normal	0.94 (0.89, 1.00)	1.05 (0.98, 1.12)
Not performed	1.09 (1.06, 1.13)	0.91 (0.87, 0.95)
**Type of TB (pulmonary)**		
Extrapulmonary	0.81 (0.75, 0.87)	1.38 (1.28, 1.48)
Both	0.66 (0.63, 0.79)	0.76 (0.72, 0.80)

DOT = directly observed therapy. HIV = human immunodeficiency virus. CI = confidence interval. Adjusted risk ratios estimated from a regression model including all exposure variables.

## Discussion

In this study we examined the relationship between treatment outcomes and individual demographics, pre-existing conditions, health-related behaviors, membership of special populations, clinical examination results, and features of health services among individuals treated for TB in Brazil between 2015 and 2018. These analyses revealed elevated risks of unsuccessful TB treatmen associated with a range of demographic, clinical and behavioral factors.

In terms of socio-demographic and behavioral factors, the strongest relationships with unsuccessful treatment outcomes were estimated for old age, no education or limited education, HIV infection, illicit drug use, and homelessness. Elevated mortality on treatment was found to be the primary cause of poor treatment outcomes for individuals with HIV and old age, while elevated loss to follow-up was the most important factors for homeless individuals and those with illicit drug use. Both factors were found to be important for individuals with no education or limited education. These findings are consistent with previous systematic reviews and meta-analyses [11–13], and point to the greater challenges of achieving successful treatment outcomes for medically fragile individuals, and for individuals with vulnerable circumstances or health behaviors that make it more difficult to complete the extended treatment regimens required for TB disease. Treatment completion was found to be higher among incarcerated patients, consistent with earlier studies [12, 14, 15]. However, TB treatment completion among incarcerated individuals may be negatively impacted when patients are transferred between facilities or released during treatment, as coordination of care is often challenging [15]. For individuals with diagnosed diabetes, we estimated a lower risk of unsuccessful treatment outcomes. This finding is in conflict with earlier studies that have reported worse treatment outcomes for individuals with diabetes [16]. In our study, it is possible that the subset of individuals with diagnosed diabetes could represent a group that was healthier and with better healthcare access compared to the overall diabetic population, and that different results may have been obtained if the diabetic category also included individuals with undiagnosed diabetes.

In terms of clinical factors, our results revealed a strong relationship between the risk of unsuccessful treatment outcomes and enrollment in DOT. Individuals who enrolled in DOT were substantially more likely to experience a successful treatment outcome, and DOT treatment was associated with lower risks of both loss to follow-up and death on treatment. It is possible these relationships are not consistent across Brazil, as the approach to providing DOT differ at the state level [17, 18]. The greater success rates experienced with DOT treatment must be interpreted carefully, as it will reflect both the impact of DOT through supporting better treatment adherence and completion (the causal effect), as well as differences in treatment outcomes resulting from differences in the characteristics of patients enrolled versus not enrolled in DOT (the non-casual effect). However, the large magnitude of this effect demonstrates the importance of DOT enrollment in understanding TB treatment outcomes in this setting. This is also shown in the results for the variable importance analysis, which found DOT to be the most important single factor for predicting treatment outcomes in this study population. As traditional DOT requires patients to consume drugs on-site multiple times per week, this can cause challenges for some patients (particularly those in vulnerable situations) and limit the proportion of patients enrolled in DOT. To address this challenge, the Brazilian health system is considering alternative DOT modalities that do not require in-person attendance (e.g., video-based DOT). If successful, these new DOT modalities could raise DOT enrollment and enhance treatment adherence (particularly in groups with currently low rates of treatment success), as well as giving patients greater autonomy over when and where they take their medication. However, it is unclear whether video DOT will meet the needs of individuals with low digital access or literacy. Additional resources and strategies may be required for these groups.

The health system level at which TB treatment is provided was also found to be strongly related to the risk of unsuccessful treatment. Controlling for other factors, patients treated in primary facilities were less likely to experience an unsuccessful treatment outcome compared to those treated in secondary or tertiary facilities. As higher-level clinical facilities typically treat individuals with more complex disease cases, it is likely the results for this variable reflect differences in case-mix between health system levels, not sufficiently captured by the other variables included in the analysis [9]. However, the high levels of unsuccessful outcome experienced by patients at higher-level facilities indicates the potential for greater absolute improvements in outcomes in these settings.

This study revealed substantial variation in treatment outcomes between states. While these differences were partially explained by inter-state variation in the patient-level factors examined in the analyses, large differences remained after controlling for these factors. Rio De Janeiro, Rio Grande do Sul, Paraná, Mato Grosso do Sul, and Roraima each had adjusted odds of unsuccessful treatment > 25% greater than the reference, while Acre, Piauí, and Rio Grande do Norte had adjusted odds of unsuccessful treatment > 25% lower than the reference. Additional studies are needed to understand the factors determining differences in treatment outcomes across states. When analyses were stratified by year, we found the estimated relationships to be generally stable over time, although ORs appeared to be declining for individuals with HIV.

Several previous studies conducted in low- and middle-income countries have focused on specific factors associated with the TB treatment outcome, such as HIV co-infection, TB drug resistance, and social vulnerability [19–21]. Our study adds to this literature by using national registry data to identify the patient subgroups that are at greater risk of poor treatment outcomes. Strengths of this study include the large sample size—allowing precise inferences—and the wide range of clinical and demographic factors available for analysis. However, this study has several limitations. Most importantly, the relationships estimated in this analysis represent statistical associations rather than causal relationships. As a consequence, while the results can be used to describe patient subgroups that are at high risk of poor outcomes—and that would potentially benefit from greater clinical attention—they do not describe the improvements in outcomes that could be achieved by changes in patient care, such as by devolving more TB care to the primary facilities or increasing DOT enrollment. Second, the outcome examined (treatment success) has limitations as an indicator of treatment effectiveness. In particular, some individuals coded as treatment success will not have achieved sterilizing cure and will go on to relapse in the years following treatment. While these relapse cases may be identified in research cohorts, they are not linked to the original treatment episode in the disease registry data. Third, we did not investigate interactions between exposure variables, or how the estimated relationships varied across states. Given the differences in TB care and populations characteristics across Brazil, it is possible such variation exists. Finally, the analysis revealed some unexpected relationships that are difficult to explain with available data (for example, the better treatment outcomes estimated for TB with both pulmonary and extrapulmonary involvement). Understanding these findings will require additional research.

## Conclusion

The fraction of patients experiencing unsuccessful TB treatment varies systematically as a function of socio-demographic factors, co-morbidities, health-related behaviors, clinical presentation, and features of clinical of care. Focusing clinical attention on patients with these risk factors could improve overall program performance and reduce disparities in treatment outcomes between population groups. Future research is needed to develop scalable treatment modalities that support regimen adherence and treatment completion, particularly among population groups with life circumstances that make this challenging.

### Electronic supplementary material

Supplementary material

## Data Availability

All data used in this study are publicly available. Data on individuals with notified TB can be accessed at the Ministry of Health of Brazil website, https://datasus.saude.gov.br/. Data on health system level of treatment locations are available from the Ministry of Health of Brazil website, https://dados.gov.br/dados/conjuntos-dados/cnes-cadastro-nacional-de-estabelecimentos-de-saude.
