# Supplementary material for: Predictors of unsuccessful tuberculosis treatment outcomes in Brazil: an analysis of 259,484 patient records
Source: BMC Infect Dis. 2024 May 27;24:531. doi: 10.1186/s12879-024-09417-7 (PMC11129366; doi:10.1186/s12879-024-09417-7)
Supplement: Supplementary file 1 — Supplementary material [file 12879_2024_9417_MOESM1_ESM.docx]

**SUPPLEMENTARY TABLE**

**Table S1. Unadjusted and adjusted odds ratios for unsuccessful treatment outcome at state level, 2015–2018.**

| State | Unadjusted odds ratio  (95% CI) | Adjusted odds ratio  (95% CI) |
| --- | --- | --- |
| Acre | 0.38 (0.31, 0.47) | 0.58 (0.47, 0.71) |
| Piauí | 0.83 (0.74, 0.94) | 0.73 (0.64, 0.83) |
| Rio Grande do Norte | 1.17 (1.08, 1.27) | 0.74 (0.68, 0.82) |
| Bahia | 1.11 (1.05, 0.98) | 0.76 (0.72, 0.80) |
| Alagoas | 1.15 (1.05, 1.26) | 0.81 (0.73, 0.89) |
| Distrito Federal | 1.09 (0.93, 1.27) | 0.86 (0.73, 1.02) |
| Pará | 0.97 (0.89, 1.05) | 0.87 (0.83, 0.92) |
| Espírito Santo | 1.06 (1.01, 1.11) | 0.88 (0.80, 0.96) |
| Paraíba | 1.24 (1.13, 1.36) | 0.89 (0.81, 0.99) |
| Minas Gerais | 1.28 (1.22, 1.34) | 0.94 (0.89, 0.99) |
| Goiás | 1.45 (1.33, 1.57) | 0.99 (0.91, 1.09) |
| São Paulo (reference) | --- | --- |
| Maranhão | 1.15 (1.08, 1.22) | 1.00 (0.94, 1.07) |
| Sergipe | 1.25 (1.19, 1.30) | 1.01 (0.91, 1.13) |
| Mato Grosso | 1.41 (1.34, 1.48) | 1.03 (0.94, 1.12) |
| Amazonas | 1.25 (1.13, 1.38) | 1.03 (0.97, 1.08) |
| Pernambuco | 1.33 (1.23, 1.43) | 1.04 (0.99, 1.10) |
| Tocantins | 0.98 (0.79, 1.21) | 1.07 (0.84, 1.35) |
| Amapá | 0.88 (0.72, 1.07) | 1.08 (0.87, 1.33) |
| Rondônia | 1.17 (1.05, 1.31) | 1.14 (1.01, 1.28) |
| Ceará | 1.25 (1.19, 1.16) | 1.19 (1.13, 1.26) |
| Santa Catarina | 1.13 (1.06, 1.21) | 1.21 (1.12, 1.30) |
| Rio De Janeiro | 1.41 (1.36, 1.45) | 1.27 (1.23, 1.32) |
| Rio Grande do Sul | 1.78 (1.71, 1.85) | 1.33 (1.27, 1.39) |
| Paraná | 0.97 (0.91, 1.04) | 1.34 (1.25, 1.44) |
| Mato Grosso do Sul | 1.50 (1.38, 1.63) | 1.35 (1.23, 1.48) |
| Roraima | 1.24 (1.02, 1.50) | 1.67 (1.35, 2.06) |

Results ordered by adjusted odds-ratio.

**Table S2. Adjusted odds ratios for unsuccessful treatment outcome stratified by year, 2015–2018.**

| **Variables (reference)** | **Adjusted OR**  **(95% CI)** | **Adjusted OR**  **(95% CI)** | **Adjusted OR**  **(95% CI)** | **Adjusted OR**  **(95% CI)** |
| --- | --- | --- | --- | --- |
|  | **2015** | **2016** | **2017** | **2018** |
| **Age (25–34)** |  |  |  |  |
| 0–4 | 0.45 (0.35, 0.57) | 0.56 (0.45, 0.70) | 0.50 (0.40, 0.63) | 0.49 (0.39, 0.60) |
| 5–14 | 0.40 (0.32, 0.49) | 0.34 (0.27, 0.43) | 0.38 (0.31, 0.47) | 0.46 (0.38, 0.55) |
| 15–24 | 1.05 (0.97, 1.12) | 1.02 (0.95, 1.09) | 1.04 (0.98, 1.12) | 1.04 (0.98, 1.11) |
| 35–44 | 1.00 (0.94, 1.07) | 0.96 (0.89, 1.02) | 0.95 (0.90, 1.01) | 0.90 (0.84, 0.96) |
| 45–54 | 0.92 (0.85, 0.99) | 0.93 (0.86, 1.00) | 0.90 (0.96, 0.96) | 0.88 (0.82, 0.95) |
| 55–64 | 1.00 (0.93, 1.09) | 1.01 (0.93, 1.09) | 0.96 (0.89, 1.04) | 0.90 (0.84, 0.98) |
| 65–74 | 1.34 (1.22, 1.48) | 1.32 (1.20, 1.46) | 1.22 (1.11, 1.34) | 1.22 (1.12, 1.34) |
| 75–84 | 1.96 (1.73, 2.22) | 2.03 (1.80, 2.29) | 1.75 (1.56, 1.97) | 1.76 (1.56, 1.98) |
| 85+ | 2.53 (2.04, 3.13) | 3.67 (3.02, 4.47) | 2.84 (2.32, 3.47) | 2.91 (2.41, 3.52) |
| **Sex (Male)** |  |  |  |  |
| Female | 0.79 (0.75, 0.83) | 0.82 (0.78, 0.86) | 0.80 (0.76, 0.84) | 0.75 (0.72, 0.79) |
| **Race (White)** |  |  |  |  |
| Black | 1.24 (1.16, 1.33) | 1.29 (1.20, 1.38) | 1.26 (1.18, 1.35) | 1.29 (1.21, 1.38) |
| Yellow | 0.91 (0.69, 1.19) | 1.00 (0.72, 1.36) | 1.05 (0.82, 1.33) | 1.07 (0.84, 1.35) |
| Mixed | 1.12 (1.07, 1.18) | 1.17 (1.11, 1.24) | 1.10 (1.05, 1.15) | 1.11 (1.06, 1.17) |
| Indigenous | 1.08 (0.86, 1.34) | 0.85 (0.67, 1.07) | 0.92 (0.74, 1.15) | 1.03 (0.82, 1.28) |
| Other | 0.96 (0.88, 1.05) | 0.99 (0.91, 1.08) | 1.02 (0.93, 1.11) | 1.03 (0.94, 1.12) |
| **Education (Complete high school)** |  |  |  |  |
| No education | 1.90 (1.67, 2.17) | 1.29 (1.57, 2.04) | 2.05 (1.10, 1.33) | 1.95 (1.73, 2.20) |
| Incomplete 1-4th grade | 1.76 (1.59, 1.96) | 1.71 (1.55, 1.90) | 1.77 (0.43, 0.59) | 1.74 (1.58, 1.92) |
| Complete 1-4th grade | 1.71 (1.56, 1.89) | 1.65 (1.50, 1.81) | 1.82 (0.54, 0.64) | 1.72 (1.58, 1.88) |
| Complete 5-8th grade | 1.34 (1.21, 1.48) | 1.33 (1.20, 1.46) | 1.47 (0.34, 0.47) | 1.45 (1.33, 1.59) |
| Any higher education | 0.77 (0.67, 0.88) | 0.82 (0.72, 0.94) | 0.77 (1.09, 1.21) | 0.74 (0.65, 0.83) |
| Other | 1.91 (1.74, 2.10) | 1.82 (1.66, 2.00) | 1.84 (1.68, 2.01) | 1.92 (1.77, 2.09) |
| **Diabetes (no)** |  |  |  |  |
| Yes | 0.97 (0.89, 1.05) | 0.98 (0.90, 1.07) | 0.89 (0.82, 0.96) | 0.91 (0.84, 0.98) |
| Other | 0.94 (0.83, 1.08) | 0.96 (0.84, 1.10) | 0.86 (0.75, 0.98) | 0.95 (0.83, 1.08) |
| **HIV (no)** |  |  |  |  |
| Yes | 3.22 (3.02, 3.45) | 2.97 (2.78, 3.18) | 2.68 (2.51, 2.86) | 2.60 (2.43, 2.77) |
| Other | 1.99 (1.89, 2.10) | 1.89 (1.79, 1.99) | 1.77 (1.68, 1.87) | 1.76 (1.67, 1.86) |
| **Smoking (no)** |  |  |  |  |
| Yes | 1.21 (1.13, 1.28) | 1.17 (1.10, 1.24) | 1.17 (1.11, 1.24) | 1.19 (1.13, 1.26) |
| Other | 1.00 (0.87, 1.16) | 0.89 (0.89, 1.20) | 1.29 (1.12, 1.49) | 1.29 (1.11, 1.50) |
| **Alcohol (no)** |  |  |  |  |
| Yes | 1.47 (1.38, 1.56) | 1.46 (1.37, 1.55) | 1.42 (1.34, 1.50) | 1.35 (1.27, 1.43) |
| Other | 0.98 (0.85, 1.13) | 1.10 (0.95, 1.27) | 1.00 (0.87, 1.16) | 1.01 (0.87, 1.17) |
| **Illicit drug use (no)** |  |  |  |  |
| Yes | 1.83 (1.70, 1.97) | 2.06 (1.91, 2.21) | 1.94 (1.82, 2.07) | 1.97 (1.85, 2.10) |
| Other | 1.17 (1.03, 1.34) | 1.23 (1.07, 1.42) | 1.16 (1.01, 1.34) | 1.22 (1.06, 1.40) |
| **Incarcerated (no)** |  |  |  |  |
| Yes | 0.55 (0.50, 0.61) | 0.51 (0.46, 0.56) | 0.54 (0.49, 0.58) | 0.49 (0.45, 0.53) |
| Other | 1.01 (0.82, 1.25) | 1.13 (0.87, 1.47) | 1.13 (0.89, 1.43) | 1.00 (0.78, 1.28) |
| **Homeless (no)** |  |  |  |  |
| Yes | 2.90 (2.57, 3.27) | 3.19 (2.83, 3.60) | 3.49 (3.11, 3.92) | 3.12 (2.79, 3.48) |
| Other | 0.97 (0.78, 1.21) | 0.88 (0.67, 1.17) | 0.86 (0.64, 1.16) | 0.84 (0.61, 1.14) |
| **Immigrants (no)** |  |  |  |  |
| Yes | 1.52 (1.11, 2.06) | 1.26 (0.96, 1.62) | 1.15 (0.87, 1.51) | 1.48 (1.18, 1.85) |
| Other | 1.04 (0.94, 1.15) | 0.94 (0.79, 1.11) | 0.98 (0.74, 1.28) | 1.07 (0.81, 1.42) |
| **Health unit (primary care)** |  |  |  |  |
| Secondary care | 1.21 (1.15, 1.27) | 1.14 (1.09, 1.21) | 1.17 (1.11, 1.23) | 1.24 (1.18, 1.30) |
| Tertiary care | 2.15 (2.02, 2.29) | 2.09 (1.96, 2.22) | 2.14 (2.02, 2.27) | 2.20 (2.08, 2.33) |
| Other | 0.98 (0.87, 1.11) | 1.07 (0.95, 1.21) | 0.96 (0.85, 1.08) | 1.06 (0.94, 1.19) |
| **DOT (yes)** |  |  |  |  |
| No | 2.59 (2.45, 2.75) | 2.30 (2.17, 2.43) | 2.40 (2.27, 2.53) | 2.38 (2.27, 2.51) |
| Other | 3.56 (3.35, 3.78) | 3.13 (2.95, 3.32) | 3.26 (3.07, 3.45) | 3.09 (2.93, 3.27) |
| **Bacteriological test (positive)** |  |  |  |  |
| Negative | 1.10 (1.03, 1.17) | 1.15 (1.08, 1.22) | 1.15 (1.09, 1.23) | 1.17 (1.10, 1.24) |
| Not determined | 1.32 (1.23, 1.40) | 1.37 (1.29, 1.46) | 1.30 (1.22, 1.38) | 1.33 (1.25, 1.41) |
| **Chest X-ray (suggestive of TB)** |  |  |  |  |
| Normal | 1.00 (0.91, 1.10) | 1.02 (0.93, 1.13) | 1.03 (0.94, 1.13) | 1.00 (0.92, 1.10) |
| Not performed | 1.02 (0.97, 1.08) | 1.05 (0.99, 1.11) | 1.03 (0.97, 1.08) | 1.07 (1.01, 1.12) |
| **Type of TB (pulmonary)** |  |  |  |  |
| Extrapulmonary | 1.07 (0.95, 1.20) | 1.01 (0.90, 1.14) | 1.04 (0.93, 1.17) | 1.01 (0.90, 1.13) |
| Both | 0.70 (0.65, 0.76) | 0.73 (0.68, 0.79) | 0.70 (0.65, 0.75) | 0.68 (0.63, 0.73) |
